# Spelling deficits in children with intellectual disabilities: Evidence from a regular orthography

**DOI:** 10.3389/fpsyg.2022.1065525

**Published:** 2023-01-17

**Authors:** Francesco Domenico Di Blasi, Francesca Vizzi, Maria Agatina Stimoli, Serafino Buono, Marika Iaia, Pierluigi Zoccolotti, Paola Angelelli

**Affiliations:** ^1^Unit of Psychology, Oasi Research Institute-IRCCS, Troina, Italy; ^2^Department of Human and Social Sciences, Lab of Applied Psychology and Intervention, University of Salento, Lecce, Italy; ^3^Department of Psychology, Sapienza University of Rome, Rome, Italy; ^4^Neuropsychology Unit, IRCCS Fondazione Santa Lucia, Rome, Italy

**Keywords:** spelling processes, spelling deficits, error analysis, intellectual disability, regular orthography

## Abstract

**Introduction:**

In individuals with intellectual disabilities (ID), efficient reading and writing skills promote social integration, self-autonomy, and independence. However, research has mainly focused on reading skills, while evidence on spelling skills is scarce and mostly on English-speaking subjects. In the present research project, we compared the spelling skills of children with intellectual disabilities (ID) learning in Italian, a regular orthography, to those of typically developing children matched for school level.

**Methods:**

In the first study, the performance on a Passage Dictation Test of forty-four children with ID attending regular classrooms from 4th to 8th grades (mean age = 12.16 years; SD = 1.57) were compared with controls matched for sex and grade. In the second study, a Words and Nonwords Dictation Test was administered (with stimuli varying for lexicality, orthographic complexity, regularity of transcription, and the presence of different types of phonetic-phonological difficulties) to twenty-two children with ID attending regular classrooms from 4th to 8th grades (mean age = 12.2 years; SD = 1.37) and 22 controls matched for sex and grade. In both studies, an error analysis was performed to characterize types of misspellings. Separate ANOVAs were performed on z scores.

**Results:**

Children with ID generally had a lower performance than controls. In the Passage Dictation Test, they showed a higher number of phonological (and phonetic-phonological) errors than phonologically plausible ones, indicating, as a group, predominant phonological difficulties as compared to lexical-orthographic ones. In the Words and Nonwords Dictation Test, they performed poorly on regular stimuli presenting specific types of phonetic-to-phonological difficulties (geminates, non-continuant consonants) and committed more minimal distance, context-sensitive and simple conversion misspellings. However, deficits in the orthographic-lexical procedure, as indicated by a low performance in words with unpredictable spelling, were present in a high percentage of children.

**Discussion:**

It is concluded that children with ID have significant spelling difficulties not confined to the orthographic process but also in phoneme-to-grapheme mapping that, in a regular orthography like Italian, should be acquired early and easily.

## Introduction

Adequate literacy is a prerequisite for achieving successful academic and professional outcomes. As for all people, for individuals with intellectual disabilities (ID), efficient reading and writing skills increase their possibility of being integrated into an ever more demanding society and consequently promote their self-autonomy and independence. Therefore, developing reading and writing abilities in children with ID is a requirement for educational achievement and, when interventions are required, a comprehension of the nature of their challenges can help develop more effective and personalized programs. A brief review of studies on reading and spelling skills in individuals with ID is presented below. The present study focuses on the spelling skills of Italian children with ID.

There is a consolidated tradition of studies on reading skills in people with ID. However, the results of this line of research strongly depend upon the methodological design employed, i.e., mental-age-match designs, reading-level-match designs, comparisons to normative data (standard scores computations) or chronological-age matched designs (see Johnson-Glenberg, 2008). According to research based on mental-age matching, children with ID generally performed better in reading words than controls but comparably in nonword reading (Laing et al., 2001; Klusek et al., 2015), highlighting weaker phonological skills than expected, given their cognitive abilities.

Some studies that compared children with ID to controls matched for reading-level found a selective impairment in nonword reading in children with ID (Verucci et al., 2006), supporting the idea that they may suffer from selective difficulties in phonological reading. However, also discordant data have been reported (Loveall and Conners, 2016). Furthermore, the appropriateness itself of this design has been questioned particularly if used to establish causality in cognitive studies of reading (van den Broeck and Geudens, 2012; Zoccolotti, 2020). Thus, it has been proposed that a nonword reading deficit may emerge as a spurious effect due to the presence of different developmental paths (Van den Broeck and Geudens, 2012), with nonword reading developing more slowly than word reading (Zoccolotti et al., 2009). By contrast, the few studies that employed chronologically matched samples or evaluated standardized scores failed to reveal difficulties in nonword encoding, while findings on word reading efficiency were more variable (Swillen et al., 1999; Lewandowski et al., 2007; for a review, see Di Blasi et al., 2018).

A different line of research focused on the literacy acquisition of children with different genetic syndromes often associated with an ID, such as Down Syndrome (DS) and Williams Syndrome (WS), with the aim to determine whether a cognitive deficit is exclusively the result of ID or is syndrome specific. In general, most studies have found a phonological deficit in both children with DS and WS (Boudreau, 2002; Menghini et al., 2004; Verucci et al., 2006; Hulme et al., 2012). Also, the reading skills of adolescents with WS have been reported as better than expected based on their IQ (Pagon et al., 1987). However, since different research designs yielded discordant results, it is difficult to point out the specific role of genetic syndromes, if any, over and beyond the influence of the experimental design adopted.

Overall, although there is a preponderance of evidence supporting a phonological reading deficit in individuals with ID, with nonword reading less efficient than word reading, it seems that this proposal is not strong enough to draw solid conclusions. Generally, the data indicate that, despite the challenges faced by children with ID, reading may be a relatively preserved skill for this population.

There has been considerably less research than on the spelling abilities of children with ID (Lindström and Lemons, 2021; de Magalhães et al., 2022). It is uncertain what level of written proficiency children with ID achieve since spelling is a very demanding and sensitive task that requires the integration of several cognitive abilities and can reveal a variety of residual errors that might otherwise be overlooked. For example, residual spelling errors can occur in adults with compensated dyslexia and relatives of dyslexic children, revealing unresolved problems (Wolff et al., 1996). Moreover, spelling may also offer insights into deficits in orthographic and phonological processing in literacy acquisition (McCarthy et al., 2012). This may be particularly useful in languages with regular orthographies (such as Serbian, Croatian, Czech, Spanish, German, or Italian), where the lack of critical items (such as irregular words or homophones) makes it difficult to test lexical processing in reading while in the phonological-to-orthographic direction instances of unpredictable spelling exist.

In Italian, the object of the present study, inefficiencies on lexical or semantic levels may be detected by measuring accuracy on targets with unpredictable spelling (e.g., phonological strings that may have multiple orthographic solutions). For instance, in Italian the phonemic group [kw], may be transcribed by the orthographic sequences QU or CU ([kwota] (rate) is spelled QUOTA and not *CUOTA) but also the syllables [tʃe], [ʃe], [dʒe], which may or may not require the letter I (e.g., [ʃena] (scene) is conveyed SCENA and not *SCIENA, but [ʃentsa] (science) is conveyed SCIENZA and not *SCENZA; for a more detailed description see Angelelli et al., 2004, 2010). Notably, not only errors but also kinetic online parameters (longer writing times and more pauses) are sensitive indicators in the case of words with unpredictable transcription (and context-sensitive words) as compared to spelling regular transcription words and pseudowords (Iaia et al., 2021). On the other hand, the relative regularity of transcription (i.e., high consistency of phoneme-to-grapheme associations) allows for also detecting failures in phonological analysis and/or transcoding.

Phonological competence is particularly important in regular orthographies where the main reliance on phonological (sublexical) procedure has been found to characterize spelling and reading acquisition (i.e., for Italian see Zoccolotti et al., 2009; Notarnicola et al., 2012; for cross-linguistic comparisons see Caravolas, 2004; Sprenger-Charolles et al., 2006; Babayiğit and Stainthorp, 2007, 2011; Marinelli et al., 2015). Following Patterson (1986), an acoustic-to-phonological conversion unit firstly identify and segment phonemic strings in the sublexical process. A phoneme-to-grapheme conversion process using sound-to-spelling correspondences activates the appropriate graphemes. However, along the acoustic-to-phonological analysis certain variables, such as the phonetic-acoustic quality, determine the complexity of the process. Vowels and fricatives ([f], [v], [s], [∫]), liquid ([l], [r]) and nasal ([n], [m], [ɲ], [ŋ]) consonants that are prolongable are easier to isolate and identify. Furthermore, words with consonant-vowel (CV) sequences are easier to analyze than those with consonant clusters (e.g., senso, [‘sɛnso], sense or valle, [‘val:e], valley). Incorrect spelling occurs despite unimpaired phoneme-to-grapheme conversion when the analysis of phonemic strings fails.

A common finding of Italian patients with acquired dysgraphia is intact phoneme-to-grapheme conversion abilities (single letter writing) but impaired isolation and identification of each phoneme within a phonemic string (Luzzatti et al., 2000). Relevant for the present investigation, studies on spelling abilities of Italian children with a history of language delay and dyslexia found a high sensitivity to acoustic-to-phonological variables, with significant failures on stimuli containing double consonants, non-continuant consonants and polysyllabic stimuli. Moreover, in this population, a high rate of phonological misspellings, such as substitutions of consonants and vowels differing only in one single distinctive feature and simple phoneme-to grapheme conversion errors. Overall, spelling more than reading (and particularly, a fine-grained analysis of misspellings) may reveal unresolved phonological processing deficits and residual language difficulties in individuals with atypical/delayed oral language development (Angelelli et al., 2016; see also Chilosi et al., 2009; Vizzi et al., 2022).

As for studies on spelling skills in children with ID, these have been conducted mainly in English-speaking countries and, similarly to those on reading skills, adopt different methodological approaches. For example, in a recent study (de Magalhães et al., 2022) on a relatively large sample of school-aged (9–17 years old) English-speaking children with WS had significantly lower spelling than reading standard scores, but there was also great variability among individuals. The results of another study (Howlin et al., 1998) on English-speaking adults (age 19–39) with WS found that their spelling ability was below functional literacy (mean spelling age equivalent was 7.6 years). Overall, the research has been predominantly conducted in a language with irregular orthography (English), and this limits the possibility to generalize results to regular orthographies.

Only a few studies examine spelling skills in individuals with ID learning languages with regular orthographies (Kortteinen et al., 2009; Maehler and Schuchardt, 2016). A study conducted on Finnish, a language with regular orthography, aimed to investigate the relationship between IQ and reading/spelling disabilities (and their relations with other cognitive skills) in a sample of adolescents, is particularly interesting for the present study (Kortteinen et al., 2009). Among subjects with reading/spelling disabilities, those with borderline intellectual functioning were worse on nonword spelling, highlighting a fragility in using the sublexical spelling procedure.

A study comparing Italian children with WS and DS (Varuzza et al., 2015), revealed lower performance of subjects with WS than typically developing (TD) children only in nonword spelling. This was probably a consequence of a difficulty in mastering the phoneme-to-grapheme conversion procedure and/or the related acoustic-to-phonological preliminary analysis. On the other hand, subjects with DS underperformed as compared to TD children and children with WS both in nonword and word spelling, revealing a fragility in the use of the lexical orthographic procedure together with failures along the phonological sublexical one. Interestingly, individuals with DS suffer from more severe expressive language skills than children with WS, especially concerning phonology and syntax (e.g., Vicari et al., 2001; Kent and Vorperian, 2013). Consistently, using a standardized spelling task, Byrne et al. (2002) found that participants with DS displayed worse spelling abilities and made less consistent development over a 2-year period than controls matched for reading ability at the start of the study. Interestingly, Bird et al. (2008) found that subjects with DS, but not TD controls, manifested lower writing accuracy with increasing word length. According to Angelelli et al. (2014, 2016), length is another complexity factor along the sublexical procedure because the longer the string to transcribe, the greater the possibility of errors. Although children with DS showed fragility in both the lexical and sublexical spelling processes, individuals with better working memory skills had adequate phonological awareness and a greater predisposition for learning the written language (Bird et al., 2000; Lavra-Pinto and Lamprecht, 2010).

However, the abovementioned studies only focused on the lexicality effect (i.e., better performance on word vs. nonword spelling) but did not investigate the efficiency of the lexical and sublexical spelling procedures by exploiting, on the one hand, irregular word spelling, and, on the other, the effect of the various sources of acoustic-to-phonological difficulties. Moreover, quantitative analysis was never corroborated by an analysis of the type of errors. The efficiency of spelling may be better probed using stimuli varying for the presence of lexical and sublexical difficulties and a fine-grained error analysis (differentiating minimal distance errors, suggestive of acoustic-to-phonological conversion failures, from simple conversion error, indicating phoneme-to-grapheme association errors and/or inefficiencies of buffering mechanisms) may shed light on the possible locus of the spelling difficulties.

In the present research project, we analyzed the spelling skills of children with mild or borderline ID and compared them to those of TD children matched for school level. In the first study, we analyzed the spelling performance on a Passage Dictation Test. In the second study, a word and nonword dictation test was administered. Single-word and-nonword spelling tasks are widely used in research and clinical settings because they allow studying the impact of known psycholinguistic effects on spelling performance. However, the Italian guidelines for the diagnosis of spelling disorders emphasize the importance of taking multiple samples of writing, and the passage dictation task may be informative for various reasons (Istituto Superiore di Sanità, 2011). A passage dictation is an ecological task for students who, in their school activities, are frequently called to write texts. In the early years of schooling, passage dictation tasks are frequent; later, dictation of arithmetic/geometric problems, assignments, and notes may occur. Even text compositions, despite involving higher-order processes, necessarily imply the process of transcription, which, if defective despite the presence of a meaningful context, may have a detrimental impact on compositional skills. Finally, in writing a passage, diverse error types may occur as compared to single-word (or-nonword) spelling: lexical spelling inefficiencies can manifest in the failure to respect the word units with erroneous fusions or segmentations of words or with substitutions between non-homographic homophones, e.g., L’AGO (the needle) vs. LAGO (lake).

In a second study, words and nonwords varying for different psycholinguistic variables, were dictated. We focused on the effect of lexicality (words vs. nonwords), orthographic complexity (regular words vs. context-sensitive words), regularity of transcription (stimuli with one-sound-to-one letter correspondence vs. stimuli with unpredictable spelling), and the presence of different sources of phonetic-phonological complexities in regular words and nonwords (such as length, continuancy of sounds, and presence of geminates). In addition, in both tasks, a qualitative error analysis was carried out in other to obtain further information on the possible loci of spelling difficulties. Considering that children with ID may display language deficits of varying degrees, we also expected their spelling performance to be characterized by defective orthographic lexical acquisition along with long-lasting phonological difficulties (similar to what was observed in children with delayed language acquisition; Chilosi et al., 2009; Angelelli et al., 2016).

## Materials and methods

### First study

#### Sample

A total of 88 children participated to the first study. All forty-four children with intellectual disabilities attended regular classrooms from 4th to 8th grades (12 subjects were 4th and 5th graders and 32 subjects attended the secondary school). Mean age was 12.16 (SD = 1.57) with a range from 8.7 to 15.1. Twenty-eight were males and 16 females. All participants were referred to the Diagnostic Clinics of Oasi Research Institute of Troina (Italy). Children were admitted to the clinics between 2019 and 2022.

International Standard Classification of Education (ISCED; UNESCO Institute for Statistics, 2012) was used to classify the educational level of parents. A total of five educational groups were considered: ISCED level 0 (less than primary education), 1 (1.14%) parent; ISCED level 1 (primary education), 5 (5.68%), ISCED level 2 (lower secondary), 60 (68%) parents; ISCED level 3 (upper secondary), 20 (22.72%); and ISCED level 6 (university degree), 2 (2.27%).

Following the indications of the Diagnostic and Statistical Manual of Mental Disorder, 5th Edition (American Psychiatric Association, 2013), the participants with ID were in either the mild intellectual disability (MID) or borderline intellectual functioning (BIF) ranges based on the Full-scale IQ (FSIQ) scores of the Wechsler Intelligence Scale for Children-fourth edition (WISC-IV; Wechsler, 2004, Italian edition by Orsini et al., 2012). Twenty-one had MID and 23 BIF. The FSIQ scores of the MID subgroup ranged from 51 to 69 (*M* = 62.2, SD = 5.4); the FSIQ scores of the BIF subgroup ranged from 71 to 84 (*M* = 75.9, SD = 4.2).

Aetiology was unspecified for most of the sampled individuals. The presence of brain damage or genetic diseases was diagnosed in seven participants (16%); among these, there were children with epilepsy (*N* = 3), and genetic mutations (*N* = 4). All of the participants had language deficits of varying degrees; in some cases (five participants; 11.3%) these deficits reached levels of severity whereby a language disorder was also diagnosed in comorbidity. Support from a special education teacher was received by 65.91% of the children; furthermore, 22.73% attended speech therapy and 20.45% psychomotor therapy.

Controls were matched one-to-many for sex and grade. Their inclusion criteria were: (i) absence of certified neurodevelopmental disorders; if (ii) normal performance (within 1 SDs of the mean) at the Raven’s Colored Progressive Matrices (CPM) (according to Pruneti et al., 1996), and (iii) adequate socio-educational conditions (none of the children were reported by their teachers for socio-economic disadvantage). The mean age was 11.64 (SD = 1.07) with a range from 9.85 to 14.1. Participants with ID just tended to be older than controls [*F*_(1,87)_ = 3.0; *p* = 0.07].

Parents were informed of research activities and authorized their child’s participation by signing the appropriate consent form. The study was conducted according to the principles of the Helsinki Declaration of 1964 and its later amendments and was approved by the local ethics committee on 12 February 2020 (2020/02/12/CE-IRCCS-OASI/PA12).

#### Passage dictation test

The Passage Dictation Test was taken from the Battery for the Assessment of Orthographic Competence (BVSCO-2, Tressoldi et al., 2013), a standardized Italian battery for the evaluation of orthographic competence in primary and secondary school. There is a different meaningful passage for each school grade, from the first year of primary school to the third year of secondary school (eighth grade). We used the passages foreseen for 4th to 8th grades. The passages differ in length, content, syntactic complexity, and word frequency, depending on age/grade.

The passage was individually dictated by the examiner to each child following the participant’ rhythm of writing. There is no provision for giving explanations in advance or during dictation on words/sentences difficult to understand, postponing any explanation until the end of the test.

The correction of the test foresees to assign one error per each misspelled word (regardless of whether the word contains one or more errors) and to classify errors in three main categories as it follows^1^:

- phonological errors (inaccurate spellings *via* the sublexical routine): errors indicating difficulties in segmenting phonemes, associating phonemes to graphemes, or phonological/graphemic buffer disorders. This category includes the following error subtypes: substitutions of letters [e.g., “FENTRE” for “VENTRE” (belly)], omission or addition of letters or syllables [e.g., “TAOLO” for “TAVOLO” (table)], inversions of letters [“NI” for “IN” (in)], and errors on diagrams [e.g., “AGI” for “AGHI” (needles)];

- phonologically plausible errors (impaired spellings along the lexical route): misspellings that sound like the target words. This category includes the following error subtypes: separations of words [e.g., “BAM BOLA” for “BAMBOLA” (doll)], fusions of words [e.g., “ILPESCE” for “IL PESCE” (the fish)], substitutions between homophonic non-homographic segments [e.g., “SQUOLA” for “SCUOLA” (school)], and omissions or additions of the silent letter “H” [HO (I have) vs. O (or) or “*HA SCUOLA” for “A SCUOLA” (at school)].

- stress errors and errors on geminates: errors in which subtle phonetic-to-phonological features are lost, such as the presence of a doubled consonant or doubled consonants that are dedoubled [e.g., “BELA” for “BELLA” (beautiful); “MARRE” for “MARE” (sea)], stress omissions or insertions [e.g., “PERCHE” for “PERCHÉ” (why)].

Finally, other types of errors were evaluated although they do not enter the total error score because not specifically related to the lexical or phonological spelling transcoding procedures. These were: (i) minor errors related to the application of written conventions (e.g., the use of capital vs. lower case letters, way of heading); (ii) the presence of correct but non dictated words; and (iii) the omission of dictated words. These errors provide information on the written competence and adherence to the delivery of the task.

#### Data analysis

The written production of each participant with ID and control participant was coded for the presence of errors and raw scores were transformed into z scores based on reference data (Tressoldi et al., 2013). Z-score data allowed us to evaluate the degree of impairment with respect to age-matched reference data (expected values = 0 and SD = 1).

An ANOVA was performed with group (participants with ID and control participants) as unrepeated factor and type of error (phonological, phonologically plausible, and phonetic-phonological errors) as repeated factor. Separate one-way ANOVAs were performed on z scores of minor errors, insertions/substitutions, or omissions of words. Finally, analyses were conducted to examine the effect of ID severity by comparing children with MID and BIF. The ANOVAs described above were carried out again with ID sub-group (MID and BIF) as unrepeated factor.

#### Results

**Figure 1:**
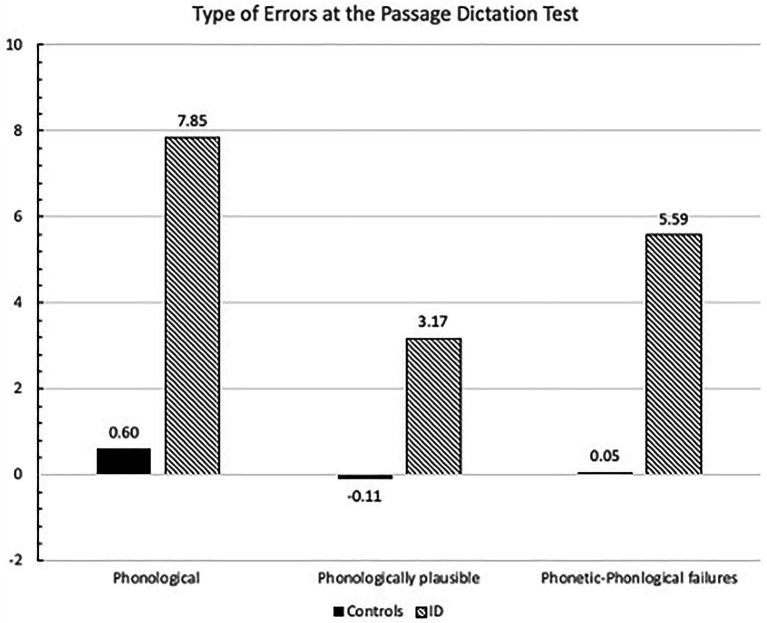
Mean error z scores for the three categories of errors at the Passage Dictation Test committed by control and ID children.

The ANOVA with group (ID and control participants) as unrepeated factor and error category (phonological, phonologically plausible and phonetic-phonological errors) as repeated factor showed a significant effect of group [*F*_(1.86)_ = 52.48; *p* < 0.0001], indicating lower performance in children with ID (5.5) than in control children (0.4), error type [*F*_(2,72)_ = 9.20; *p* < 0.0001], with phonological errors being higher than phonologically plausible ones (4.4 vs. 1.5, respectively; *p* < 0.001). The group x error type interaction was significant [*F*_(2,172)_ = 5.05; *p* < 0.01; see Figure 1]: children with ID had generally lower performance for all types of errors with respect to control children (at least *p* < 0.01 in all comparisons), but simple effects within the group with ID showed that they presented higher number of phonological errors with respect to phonologically plausible ones (*p* < 0.0001); also, phonetic-phonological errors tended to be higher than phonologically plausible ones (*p* = 0.08). In controls, only small deviations from normative means were evident and no significant differences emerged between types of errors.

The ANOVA with ID sub-group (MID and BIF) as unrepeated factor showed a significant effect of error type [*F*_(2,42)_ = 7.42; *p* < 0.0001], with phonological errors being higher than phonologically plausible ones (7.8 vs. 3.17, respectively; *p* < 0.001). The group x error type interaction [*F*_(2,84)_ = 5.05; *p* < 0.01] indicated that children with MID had higher rates of phonologically plausible errors than children with BIF (4.8 vs. 1.6; *p* < 0.05), while the two sub-groups showed comparable rates of phonological errors (8.1 and 7.6, respectively) and phonetic-phonological errors (4.0 vs. 7.1, respectively).

The ANOVA on minor errors showed that children with ID as well as controls presented only marginal deviations from the normative values [0.28 vs. 0.04; *F*_(1,87)_ = 0.70; n.s.], showing adequate knowledge of written conventions. Similarly, children with ID did not omit more words than control children [0.03 vs. 0.2; *F*_(1,87)_ = 0.01; ns] and introduced new (not dictated) words in the passage more rarely than did control children [−0.03 vs. 0.24; *F*_(1,87)_ = 6.71; *p* < 0.01]. Both the latter indices denote good adherence to the task in children with ID.

No differences emerged between children with MID and BIF in the rates of written conventions errors [*F*_(1,43)_ = 1.51; ns], presence of correct but non-dictated words [*F*_(1,43)_ = 0.82; ns] or word omissions [*F*_(1,43)_ = 0.18; ns].

### Second study

#### Sample

A total of 44 children participated in the second study. Twenty-two children with ID, all attending regular classrooms from 4th to 8th grades (5 children were 4th and 5th graders, and 17 attended secondary school). Seventeen of the 22 children with ID participated also the first study (while five participants were new children). The mean age was 12.2 (SD = 1.37) with a range from 9.9 to 14. Ten were males and 12 were females. All participants were referred to the Diagnostic Clinics of Oasi Research Institute of Troina (Italy). Children were admitted to the clinics between 2019 and 2022.

International Standard Classification of Education (ISCED; UNESCO Institute for Statistics, 2012) was used to classify the educational level of parents. Five educational groups were considered: ISCED level 0 (less than primary education), 0 of parents fell in this category; ISCED level 1 (primary education), 3 (7%) of parents; ISCED level 2 (lower secondary), 29 (66%) of parents; ISCED level 3 (upper secondary), 12 (27%); and ISCED level 6 (university degree), 0.

According to the Diagnostic and Statistical Manual of Mental Disorder, 5th Edition (American Psychiatric Association, 2013), the participants were in the MID (*N* = 12) or BIF (*N* = 10) ranges based on the FSIQ scores of the WISC-IV The FSIQ scores of the MID subgroup ranged from 53 to 67 (*M* = 60.7, SD = 5); the FSIQ scores of BIF subgroup ranged from 71 to 77 (*M* = 74.5, SD = 2.8). Aetiology was unspecified for most of the sampled individuals. The presence of brain damage or genetic diseases was diagnosed in four participants (18%); among these, there were children with epilepsy (*N* = 2), and genetic mutations (*N* = 2); four participants (18%) showed language impairment. Support from a special education teacher was received by 77.27% of children; furthermore, 9.09% attended speech therapy and 4.55% attended psychomotor therapy.

Controls were matched one-to-many for sex and grade. Their inclusion criteria were: (i) absence of certificated neurodevelopmental disorders; if (ii) normal performance (within 2 SD of the mean) at the Raven’s CPM (according to Pruneti et al., 1996), and (iii) adequate socio-educational conditions (none of the children were reported by their teachers for socio-economic disadvantage). The mean age was 12.24 (SD = 1.43) with a range from 8.7 to 14.17. Participants with ID and controls were of comparable age [*F*_(1,42)_ < 1; n.s.].

Parents were informed of research activities and authorized their child’s participation by signing the appropriate consent form.

#### Word and nonword dictation test

Spelling abilities were tested through a standard single Word and Nonword Dictation Test (Angelelli et al., 2016; see Appendix A), composed of four sections:

Section A: regular words with complete correspondence between sounds and letters (*N* = 70). To determine the variables influencing segmentation and identification of phonemic strings to be converted, different sources of phonetic-phonological complexity were used. In particular, we selected the words as follows: (i) words consisting only of continuant sounds (fricative, liquid, nasal consonants) versus words containing non-continuant (plosive) consonants; (ii) words consisting only of consonant-vowel syllables and consonant clusters; and (iii) disyllabic words versus polysyllabic words.

Section B: regular words that require context-sensitive sound-to-spelling rules (*N* = 10). In Italian, context-sensitive rules are required when the spelling of a consonant depends on the following sound. For instance, the phoneme [k], is spelled C when followed by a consonant (e.g., CLIMA [klima], climate) or by A, O, U (e.g., CASA [kaza], home) and CH when followed by E or I (e.g., BARCHE [barke], boats).

Section C: unpredictable transcription words along phonological-to-orthographic conversion routine (*N* = 55). This section includes the following categories: (i) words with the phonemic group [kw], which in Italian may be transcribed by orthographic sequences QU, CU, or CQU; (ii) words containing syllables [t∫e], [∫e], [dʒe], which may or may not require an I (e.g., [∫entsa], science, is spelt SCIENZA and not *SCENZA, while [∫ena], scene, is spelt SCENA and not *SCIENA); (iii) words containing plosive phones followed by liquid consonants [r] which are homophones to their doubled pairs (e.g., FEBBRE, fever and not *FEBRE, but LIBRO, book, and not *LIBBRO); (iv) words containing segments [lj] – [ʎ] and [nj] – [ɲ], that are homophonous in most Italian variants to the extent that [biljardo/biʎardo], billiards, is spelt BILIARDO and not *BIGLIARDO, while [folja/foʎa], leaf, is spelt FOGLIA and not *FOLIA; similarly [opinjone/opiɲone], opinion, is spelt OPINIONE and not *OPIGNONE, while [oɲuno/onjuno], everyone, is spelt OGNUNO and not *ONIUNO.

Section D: nonwords with complete correspondence between sounds and letters (*N* = 25). Different types of phonetic-phonological complexity were controlled for items, as well as for words in Section A: (i) continuance of sounds (nonwords with continuant versus non-continuant consonants), syllabic structure (nonwords with consonant-vowel (CV) syllables versus nonwords also containing doubled consonants), and length (disyllabic versus 3–4 syllable nonwords). Like Section A, phonetic/phonological variables are introduced to account for variables influencing acoustic-to-phonological analysis that is preliminary to an effective phonological-to-orthographic conversion procedure.

Words and nonwords were given in separate sequences and in a single quasi-randomized order. Children were examined individually. Each item was read aloud in a neutral tone, i.e., without highlighting clusters, doubled consonants, or possible orthographic ambiguities. Before writing down each item, children were asked to repeat it (so the examiner could make sure they understood the item). Upon request or failure to repeat, the examiner read the stimulus again to the children. There was a very low rate of errors (about 1% of cases), and the second repetition was always sufficient to obtain a correct response. Both capital and lower-case letters were allowed. Feedback on accuracy of written responses was not provided. Final responses were counted, irrespective of correctness of first attempt.

The test has normative data from the 1st to the 8th grade (Angelelli et al., 2016).

### Data analysis

#### Quantitative analysis

Firstly, the number of correct spellings on each of the four sections of test was counted for every participant with ID and control participant. We then computed the z scores for each section of the test following the reference data (Angelelli et al., 2016). Moreover, to specifically evaluate efficiency of phonetic-to-phonological analysis, we computed for each participant the number of correct responses in the various subsets of words (Section A, sub-sets 1–7) and nonwords (section D, sub-sets 1–5) transformed into z scores.

The first ANOVA was performed on total spelling accuracy score, with group (ID and control participants) as unrepeated factor. A second ANOVA was performed on type of stimuli (regular words, context-sensitive words, unpredictable words, and nonwords) as within-factor and group (ID and control participants) as unrepeated factor.

In a third ANOVA, the effect of phonetic-to-phonological difficulties on regular words and nonwords spelling accuracy was analyzed between the two groups. In this analysis, the group (ID and control participants) entered as unrepeated factor, while lexicality (words, nonwords) and type of phonetic-to-phonological difficulties (3 levels: length: disyllabic (short) vs. polysyllabic (long) stimuli; presence of geminate: single vs. doubled consonants; continuance of sounds: continuant vs. occlusive sounds) as repeated factors. Regarding phonetic-to-phonological difficulties, the ANOVA examined the effect of:

- continuance of sounds (stimuli with continuant versus non-continuant consonants). Operationally for words, we compared z scores of subsets 1 + 3 + 4 vs. 5 + 6 + 7; for nonwords, subsets 1 + 3 vs. 4 + 5;

- length (disyllabic versus polysyllabic stimuli). Operationally for words, we compared z scores of subsets 1 vs. 2; for nonwords, subsets 1 vs. 2;

- the presence of geminate consonants [stimuli made up of consonant-vowel (CV) syllables vs. stimuli containing doubled consonants]. Operationally for words, we compared z scores of subsets 1 vs. 4 and 5 vs. 7; for nonwords, we contrasted subsets 1 vs. 3 and 4 vs. 5.

The three ANOVAs described above were also performed with ID sub-group (MID and BIF) as unrepeated factor.

Interactions were explored by: (1) t-tests comparing groups on the various sections/subsets; (2) *t*-tests for paired samples, exploring simple effects within the groups.

### Qualitative error analysis

An analysis was performed to identify the nature of spelling errors, irrespective of the section of the test in which they emerged. Based on previous studies (Angelelli et al., 2004, 2010, 2016), errors were coded as:

Phonologically plausible errors (impaired spellings along the lexical route): misspellings that sound like the target words; these errors arise from over-reliance on phoneme-to-grapheme conversion routine [e.g., *CUOTA instead of QUOTA, (rate); *FEBRE instead of FEBBRE, (fever) and the other instances described in Section C of the spelling assessment];

Phonological errors (inaccurate spellings *via* sublexical routine): errors indicating difficulties in segmenting phonemes, associating phonemes to graphemes, or phonological/graphemic buffer disorders. This category included the following error subtypes:

Errors based on minimal distance (MD) features: substitutions of consonants or vowels with other consonants or vowels that differs only in one single distinctive feature [e.g., sonority, FINO (until) instead of VINO (wine); continuance, PESTA (crush) instead of FESTA (holiday)]. Doubling of a single consonant or dedoubling of a doubled consonant were also considered in this category;

Other errors: non-minimal-distance substitutions [e.g., *BALO instead of BACO (worm)], omissions [e.g., *VSONE instead of VISONE (mink)], insertions [e.g., *MANRMO instead of MARMO (marble)] and letter transpositions [e.g., *PATRO instead of PRATO (field)];

Context-sensitive sound-to-spelling errors: errors in the application of context-sensitive sound-to-spelling rules [e.g., *ADAGO instead of ADAGIO (slow) or *SCEDA instead of SCHEDA (card)].

Also in this case, errors were transformed into z scores following the reference data (Angelelli et al., 2016). An ANOVA was carried out with group (ID and control participants) as unrepeated factor and error type (phonologically plausible, minimal distance, other errors, and context-sensitive sound-to-spelling errors) as repeated factor. A second ANOVA was performed to compare the error profile of the two groups (ID and control participants) with errors collapsed into lexical vs. non-lexical categories.

Two similar ANOVAs examined the effect of ID severity on the error profile of the two groups of children with ID; in this ANOVAs, the ID sub-group (MID and BIF) was the unrepeated factor. In one analysis, four error types were considered (phonologically plausible, minimal distance, other errors, and context-sensitive sound-to-spelling errors); in the other, errors were collapsed into lexical vs. non-lexical categories.

In all cases interactions were explored by: (1) *t*-tests comparing groups on the various categories of errors; (2) *t*-tests for paired samples, exploring simple effects within the groups.

### Results

#### Quantitative analysis

**Figure 2:**
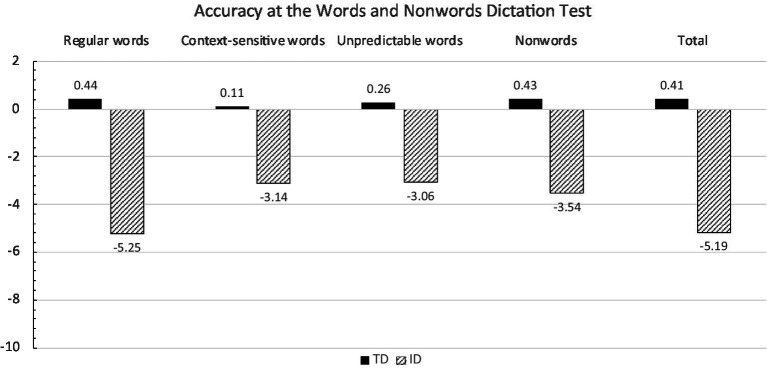
Mean accuracy z scores in the total and in the four sections of Words and Nonwords Dictation Test by control and ID children.

Figure 2 reports mean accuracy z scores for children with ID and control participants in the four sections of the test and in the whole test, respectively.

The ANOVA on total accuracy scores showed a significant effect of group [*F*_(1,43)_ = 33.83; *p* < 0.0001]: children with ID were significantly worse than controls (−5.19 vs. 0.41, respectively).

In the ANOVA with group and type of stimuli (regular words, context-sensitive words, unpredictable words, and nonwords) the main effects of group [*F*_(1,42)_ = 35.97; *p* < 0.0001] and type of stimuli [*F*_(3.126)_ = 2.94; *p* < 0.05] as well as the group by type of stimuli interaction [*F*_(3,126)_ = 4.39; *p* < 0.01] were significant. We focus on the first-order interaction. In all categories of stimuli (at least *p* < 0.0001), children with ID obtained lower performances than age-matched controls. However, intra-group comparisons showed that children with ID were worse in spelling regular words with one-sound-to-one letter correspondence with respect to unpredictable transcription words and context-sensitive words (at least *p* < 0.05), and also tended to be worse with respect to nonwords (*p* = 0.07). By contrast, no significant differences emerged between unpredictable transcription words, context-sensitive words, and nonwords. In controls, as expected, only marginal deviations from normative means were evident and no significant differences emerged between types of stimuli.

**Figure 3:**
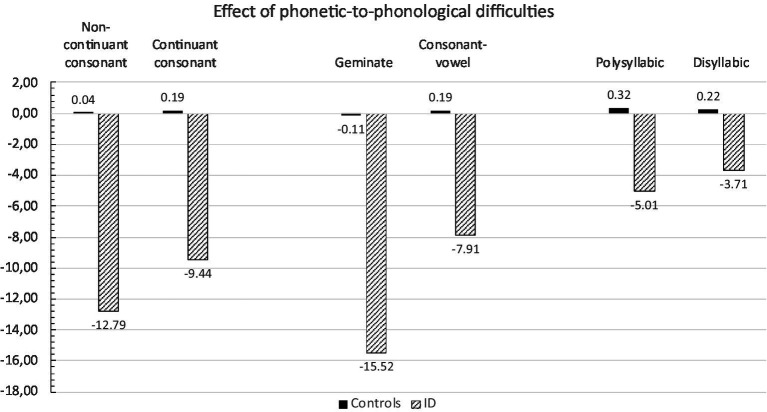
Mean accuracy z scores for the different subsets of Words and Nonwords Dictation Test by control and ID children.

The ANOVA exploring the influence of different sources of phonetic-to-phonological complexity in words and nonwords transcription showed the main effects of group [*F*_(1,42)_ = 23.75, *p* < 0.0001], with children with ID underperforming as compared to controls (−7.25 vs. 0.11, respectively), and type of phonetic-to-phonological difficulties [*F*_(5,210)_ = 7.21, *p* < 0.0001], with stimuli containing doubled consonants and long stimuli spelled worse than those without doubled consonants and short ones, respectively (*p* at least <0.05). Furthermore, the first-order group by type of phonetic-to-phonological difficulties interaction was significant [*F*_(5,210)_ = 6.33, *p* < 0.0001]: the performance of children with ID was modulated by phonetic-to-phonological difficulties to a greater extent than that of controls (for each dependent variables examined at least *p* < 0.01; see Figure 3). For children with ID, the presence of non-continuant sounds produced a significant reduction in accuracy compared to continuant sounds [*t*_(21)_ = 2.27; *p* < 0.05]; similarly, the presence of doubled consonants produced a significant reduction of spelling accuracy compared to the condition without doubled consonants [*t*_(21)_ = 2.26; *p* < 0.05]. The difference in accuracy between long and short stimuli was not significant. For controls, as expected, variations from the normative means were minimal and not significant. The main effect of lexicality was not significant [*F*_(1,42)_ = 0.59, n.s], nor were the group by lexicality [*F*_(1,210)_ = 0.71, n.s], lexicality by phonetic-to-phonological complexity [*F*_(5,210)_ = 0.17, n.s.] and lexicality by phonetic-to-phonological complexity by group [*F*_(5,210)_ = 0.33, n.s.] interactions.

In the ANOVAs comparing children with MID and BIF, there was no significant effect of the ID sub-group factor on the total accuracy score [*F*_(1,21)_ = 1.02; ns], indicating a comparable degree of impairment. The group by type of stimuli [*F*_(3,60)_ = 0.45; ns] and group by phonetic-to-phonological difficulties [*F*_(5,100)_ = 0.57; ns] first-order interactions were not significant, indicating a similar modulation of type of stimuli and phonetic-to-phonological difficulties in the two sub-groups.

### Qualitative error analysis

**Figure 4:**
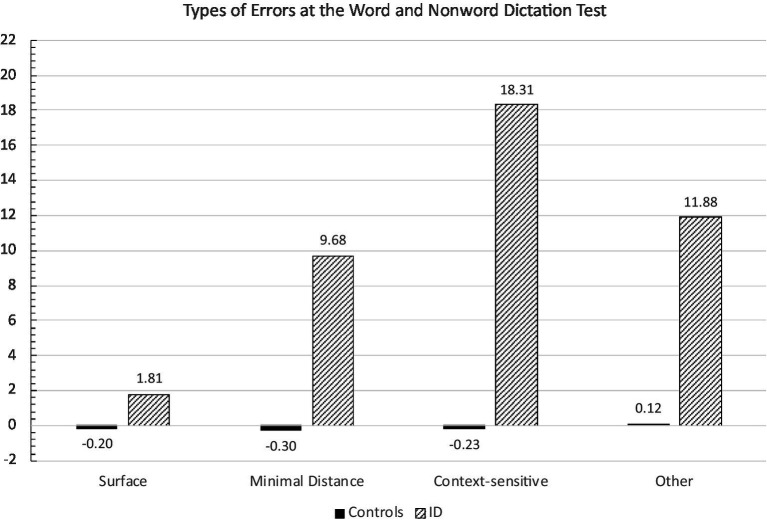
Mean errors z scores for the various categories of errors in the Words and Nonwords Dictation Test committed by control and ID children.

To better clarify the nature of the spelling deficit in the two groups, an analysis of error types was performed. The ANOVA with group (ID and control participants) and error category (phonologically plausible, context-sensitive, MD and other errors) showed a significant effect of group [*F*_(1,42)_ = 21.26; *p* < 0.0001], with children with ID presenting higher error scores than controls [10.41 vs. −0.15, respectively], error type [*F*_(3,126)_ = 3.82; *p* < 0.01], with phonologically plausible errors (0.81) being lower than MD (4.7), other (6.0), and context-sensitive errors (16.3). The group x error type interaction was significant [*F*_(3,126)_ = 3.79; *p* < 0.01]: children with ID had a higher rate of all types of errors than controls (at least *p* < 0.01 in all comparisons), but simple effects within the ID group showed that they presented lower scores for phonologically plausible errors than MD, context-sensitive and other misspellings (at least *p* < 0.01; see Figure 4). For controls only marginal and not significant deviations from normative means were evident.

**Figure 5:**
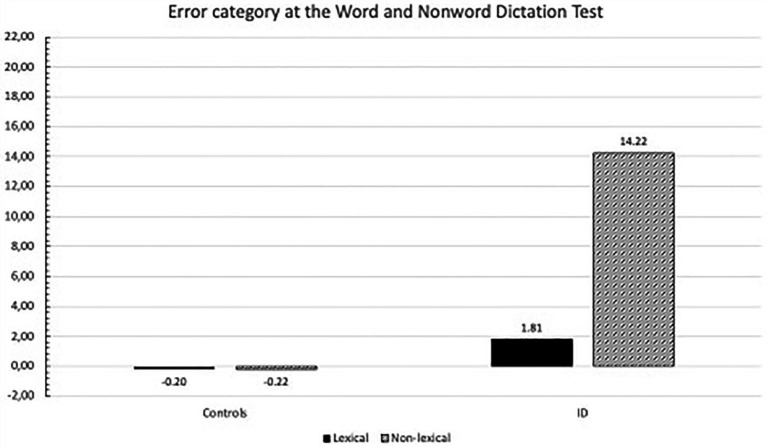
Mean z error scores for lexical and non-lexical errors in Word and Nonword Dictation Test committed by controls and ID children.

The ANOVA on errors collapsed into lexical vs. non-lexical categories showed the significant main effect of group (*F*_(1,42)_ = 22.98; *p* < 0.0001), with children with ID performing worse than controls (8.01 vs. –0.21, respectively). The group x error type interaction was significant (*F*_(1,42)_ = 14.48; *p* < 0.0001): only children with ID made significantly more non-lexical than lexical errors (*p* < 0.001; see Figure 5).

In the ANOVA comparing children with MID and BIF on the four error types (phonologically plausible, context-sensitive, MD and other errors) there were no significant differences between sub-groups [*F*_(1,20)_ = 1.87; n.s.] and also the group by error type interaction was not significant [*F*_(3,60)_ = 1.79; n.s.], indicating a comparable error profile in the two sub-groups. Similarly, when errors were collapsed into the lexical vs. non-lexical categories, no significant differences emerged between sub-groups [*F*_(1,20)_ = 54; n.s.] and the interaction group by error type was not significant [*F*_(1,20)_ = 14; n.s.].

### Individual data analysis

**Figure 6:**
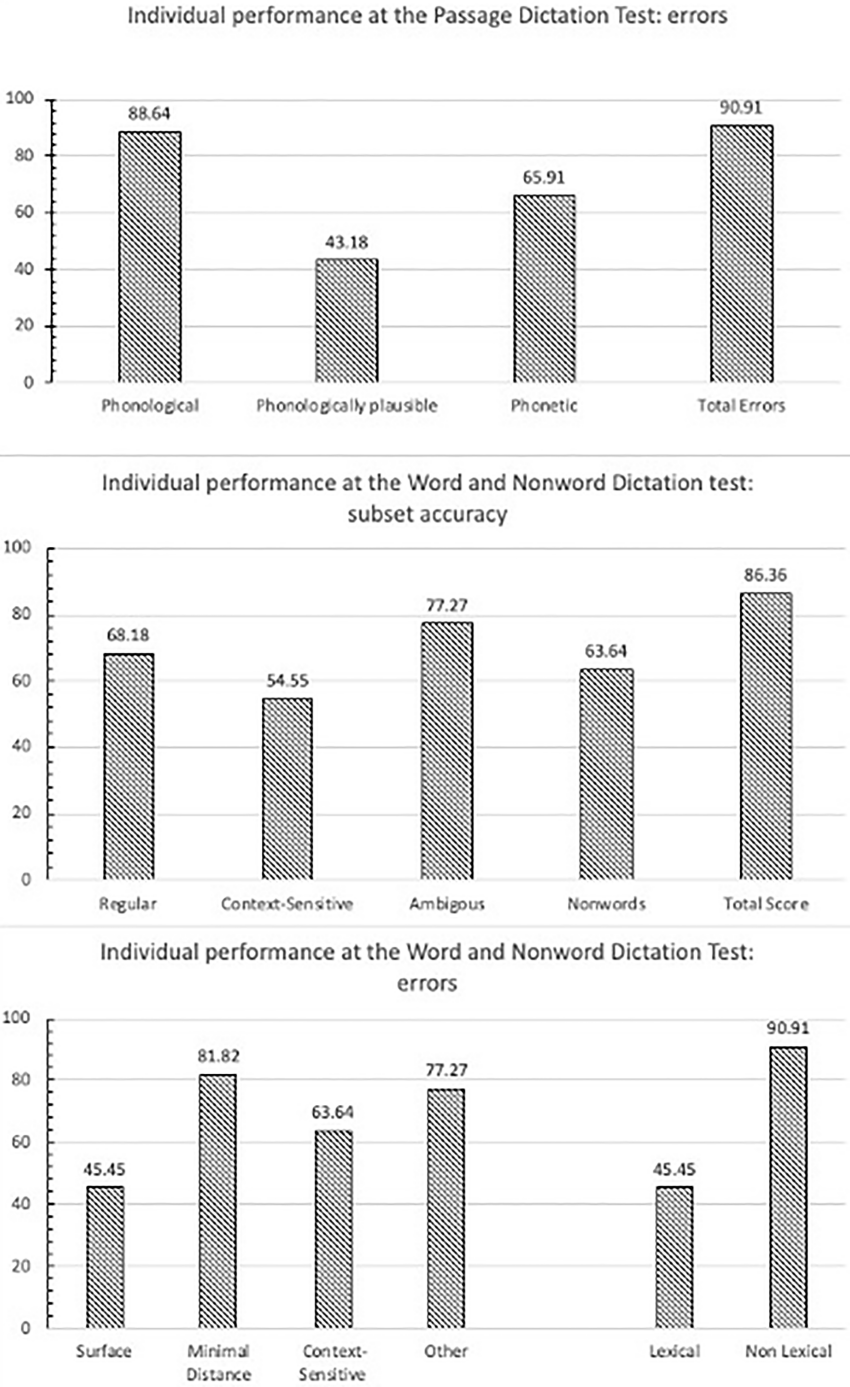
Proportion of children with ID who fell below the −1.65 cut-off for the various measures.

Individual variability in the spelling tests used in studies 1 and 2 can be appreciated in Figure 6 that reports the proportion of participants with ID who fell below the −1.65 cut-off for the various measures computed. As it can be seen, there is some degree of individual variability although not overreaching: in the total score of both spelling tests a proportion of nearly 90% of participants with ID fell below the norm. The highest variability was for surface/phonologically plausible scores where the proportion of participants underperforming was about 45% (with 55% of children displaying a proportion of lexical errors within the norm). By contrast, for the different categories of phonological errors the proportion of participant underperforming ranged from a minimum of 63% (for context-sensitivity errors) to approximately 82% (for minimal distance errors).

## Discussion

This study aimed to investigate the spelling abilities of children with ID and to characterize their pattern of spelling impairment compared to age-matched controls.

In general, children with ID were poorer spellers than controls. This finding was expected based on previous research (Byrne et al., 2002; Bird et al., 2008; Ratz and Lenhard, 2013; de Magalhães et al., 2022). However, children with ID were not simply worse than controls but presented a peculiar pattern of impairment: in both spelling tests, they displayed more pronounced phonological than lexical-orthographic difficulties. In the Passage Dictation Test, they presented more phonological and phonetic-phonological errors with respect to phonologically plausible ones. In the Word and Nonword Dictation Test, they were poorer on regular stimuli presenting specific sources of phonetic-to-phonological complexity (geminates, non-continuant consonants) and committed more minimal distance, context-sensitive and simple conversion misspellings. This does not mean that the orthographic-lexical procedure was fully efficient: 45% of children fell below the cut-off based on normative samples for surface errors and as a group, they performed worse than control participants also on unpredictable word spelling. However, the use of z scores allowed us to highlight concurrent difficulties also in the easiest conditions (i.e., regular one-sound-to-one-letter correspondence words and nonwords). Note that, when different levels of difficulty are used to compare groups of individuals with typical versus atypical development in terms of raw data, the most difficult conditions typically tend to produce greater differences among less proficient individuals over and above the specific effect of the experimental manipulation. This is known as the over-additivity effect (for a discussion, see Faust et al., 1999), and *ad hoc* data transformations, including z scores, are needed to control for it. Thus, the use of z scores proves particularly useful when levels vary in terms of general difficulty, allowing the identification of difficulties even when conditions are comparatively easy.

Overall, in addition to spelling difficulties on words with unpredictable transcription, requiring access to lexical representation, children with ID were also poorer on regular stimuli presenting specific sources of phonetic-to-phonological complexity.

Poor performance on items requiring lexical knowledge is in line with other studies on Italian typically developing children (Notarnicola et al., 2012) showing that optimization in the use of the lexical spelling procedure is reached quite slowly. The same study highlighted a ceiling effect for regular stimuli around fourth grade, indicating that, in Italian typically developing children, the conversion routines involved in sublexical processing (acoustic-to-phonological and phoneme-to-grapheme) are optimized early, i.e., within the first 3–4 years of schooling. However, difficulties in sub-lexical processing may be associated to fragilities in language acquisition. As compared to dyslexic children without oral language difficulties, those with a history of language delay presented persisting phonological spelling difficulties influenced by the phonological complexity of stimuli (especially the presence of doubled consonants but also non-continuant sounds and length; Chilosi et al., 2009; Angelelli et al., 2016). Greater spelling difficulties with stimuli containing phonetic-to-phonological difficulties are coherent with the fragility of the acoustic-to-phonological conversion unit. Non-continuant phones are more difficult to segment and identify than continuant ones. Predominant misspellings consisting of devoicing of voiced consonants and doubling of single (or dedoubling of doubled consonants) have been described in Italian brain-damaged patients with acquired dysgraphia and ascribed to acoustic-to-phonological deficits (Luzzatti et al., 2000).

Also, the error analysis produced informative results. Children with ID committed a high number of minimal distance substitutions, as well as simple conversion and context-sensitive errors. The high prevalence of these errors corroborates the results obtained in the Passage Dictation Test, which could be affected by the way the test is corrected: in the case of several errors on a word, the phonological error is assigned because it is considered more serious (Tressoldi et al., 2013). However, also the Words and Non-words Dictation Test, which allows for the assignment of all errors made on a word (without favoring the phonological ones), confirmed the high rate of phonological errors. Minimal distance misspellings indicate a particular fragility when processing minimal distinctive features sublexically. This weakness may be attributed to difficulties with phonetic/phonological analysis in children with atypical language development (Angelelli et al., 2016). Furthermore, Ziegler et al. (2005) found that children with language impairment had poorer than normal consonant identification, especially for voicing perception when measured in ecological conditions (speech perception–in-noise-measure). The authors interpreted the consonant identification difficulties in children with language impairment as due to a central deficit in feature extraction, i.e., inefficient mapping acoustic into phonetic features, rather than a deficit of low-level capacities.

Given this evidence, the analysis of spelling performance may have captured fragilities in processing subtle phonetic-to-phonological traits still present in children with ID. Moreover, the sublexical spelling procedure may suffer from working memory functioning inefficiencies, as already reported in this population (Bird et al., 2000; Lavra-Pinto and Lamprecht, 2010). In future studies, it will be interesting to investigate the relationship between previous language delays and spelling competence in this population, and to explore the role of working memory in the pattern observed.

Overall, results showed that children with ID suffered from spelling deficits, confirming the presence of literacy acquisition deficits in children with ID even in languages with regular sound-to-spelling mapping. The generally worse performance of children with ID is consistent with previous findings (Byrne et al., 2002; Varuzza et al., 2015), although comparisons with other studies are difficult due to methodological differences. The only Italian study that, to our knowledge, analyzed the spelling competence of children with ID focused on differences between syndromes (DS and WS) and used a spelling-to-dictation test of single words and nonwords not controlled for variables other than lexicality (and no error analysis was performed). However, despite these differences, Varuzza et al. (2015) found that both children with DS and WS were poorer than typically developing children in nonword spelling, revealing difficulties related to sublexical processing. Moreover, only children with DS made more errors in word spelling than children with WS and TD children as a consequence of the difficulty in using not only the sublexical spelling procedure but also the lexical-orthographic one (Vicari et al., 2001; Kent and Vorperian, 2013). In our study, the use of quantitative and qualitative analysis revealed poor lexical and sublexical spelling processes in individuals with unspecified ID, indicating, at the net of a specific syndrome, both lexical-orthographic and phonological inefficiencies.

We also examined whether there was an influence of the degree of intellectual disability over the spelling performance by comparing children with MID with children with BIF. Only in the Passage Dictation Test children with MID presented a higher rate of phonologically plausible errors than children with BIF while no influence was detected in the Word and Nonword Spelling Test. These data indicate a limited effect of ID severity on spelling performance although the division into sub-groups clearly weakened the sensitivity of our tests and further research is needed to confirm this finding.

The data from the present study have both clinical and educational implications. Clinically, the possibility of using materials controlled for the most relevant psycholinguistic variables and performing an error analysis may help to corroborate the diagnosis of spelling deficits and qualify the sources of spelling difficulties. Moreover, the characterization of spelling difficulties may allow more focused treatment. School education heavily rests on tasks requiring adequate spelling abilities, a prerequisite for the more complex compositional written abilities/tasks.

Therefore, our results represent new evidence that underscores how Italian children with ID have significant spelling difficulties not confined to the orthographic process but also to the phoneme-to-grapheme mapping, that in a regular language like Italian, should be acquired early and easily (Notarnicola et al., 2012). However, the heterogeneity of students with ID together with the multitude of other variables that influence their learning and development (such as different socio-cultural family backgrounds or additional medical diagnoses), makes the study of writing skills in children with ID particularly complex and caution warranted before generalizing the present results.

## Data availability statement

The raw data supporting the conclusions of this article will be made available by the authors, after evaluation of the purpose of the study and/or research project.

## Ethics statement

The studies involving human participants were reviewed and approved by the Local Ethics Committee Oasi Research Institute of Troina (Italy) on 12 February 2020 (2020/02/12/CE-IRCCS-OASI/PA12). Written informed consent to participate in this study was provided by the participants’ legal guardian/next of kin.

## Author contributions

FDDB and FV designed the experiment. MAS and FDDB collected the data. FV, MI, and PA performed the data analysis. SB, PZ, and PA supervised the whole study. FDDB, FV and PA prepared the first draft and all authors agreed on the manuscript.

## Conflict of interest

The authors declare that the research was conducted in the absence of any commercial or financial relationships that could be construed as a potential conflict of interest.

## Publisher’s note

All claims expressed in this article are solely those of the authors and do not necessarily represent those of their affiliated organizations, or those of the publisher, the editors and the reviewers. Any product that may be evaluated in this article, or claim that may be made by its manufacturer, is not guaranteed or endorsed by the publisher.

## Footnotes

^1^If a word contains several errors the most serious (in terms of recognisability of the target word) is counted. In this battery, phonological errors are considered more detrimental in terms of recognisability of the target than phonologically plausible errors, which are more serious than stress errors or errors on geminates.

## Appendix A Sections of the Word and Nonword spelling test (Angelelli et al., 2016).

**A**. Regular words with one-sound-to-one-letter correspondence (*n* = 70).

**Table 1:**

	Examples (translation)	Continuance	Cluster	Doubled consonants	Syllables	*N*
1	Sole (sun)	Yes	No	No	2	10
2	Lavoro (work)	Yes	No	No	3/4	10
3	Senso (sense)	Yes	Yes	No	2	10
4	Valle (valley)	Yes	No	Yes	2	10
5	Dito (finger)	No	No	No	2	10
6	Prato (field)	No	Yes	No	2	10
7	Tappo (cork)	No	No	Yes	2	10

**B**. Regular words (requiring context-sensitive sound-to-spelling rules) (*n* = 10).

**Table 2:**

	Examples	Rule	*N*
8	gola/ghiro/valigia (throat/dormouse/suitcase)	k, g, t∫, dʒ	10

**C**. Words with unpredictable transcription (*n* = 55).

**Table 3:**

	Examples	(Translation)	Ambiguity	N
9	scena/scienza	(scene/science)	[t∫e], [S∫e], [dʒe] ± i	10
10	paglia/balia	(straw/nurse)	[ʎ]: GL/LI	10
11	segno/genio	(sign/genius)	[ɲ]: GN/NI	10
12	libro/febbre	(book/fever)	BR/BBR	10
13	cuore/quota/aquila	(heart/share/eagle)	[kw]: CU/QU	15

**D**. Nonwords with one-sound-to-one-letter correspondence (*n* = 25).

**Table 4:**

	Examples	Continuance	Cluster	Doubled consonant	Syllables	*N*
1	nise	Yes	No	No	2	5
2	vimàne/ramàsola	Yes	No	No	3/4	5
3	seffa	Yes	No	Yes	2	5
4	tido	No	No	No	2	5
5	nitta	No	No	Yes	2	5

## References

[ref1] American Psychiatric Association (2013). Diagnostic and Statistical Manual of Mental Disorders, 5th Edn. Washington, DC: American Psychiatric Association.

[ref2] Angelelli P. Judica A. Spinelli D. Zoccolotti P. Luzzatti C. (2004). Characteristics of writing disorders in Italian dyslexic children. Cogn. Behav. Neurol. 17, 18–31. doi: 10.1097/00146965-200403000-00003, PMID: 15209222

[ref3] Angelelli P. Marinelli C. V. Burani C. (2014). The effect of morphology on spelling and reading accuracy: a study on Italian children. Front. Psychol. 5:1373. doi: 10.3389/fpsyg.2014.01373, PMID: 25477855 PMC4237035

[ref4] Angelelli P. Notarnicola A. Judica A. Zoccolotti P. Luzzatti C. (2010). Spelling impairment in Italian dyslexic children: does the phenomenology change with age? Cortex 46, 1299–1311. doi: 10.1016/j.cortex.2010.06.015, PMID: 20688322

[ref5] Angelelli P. Putzolu A. Iaia M. Marinelli C. V. Gasperini F. Chilosi A. M. (2016). Spelling impairments in Italian dyslexic children with and without a history of early language delay. Are there any differences? Front. Psychol. 7:527. doi: 10.3389/fpsyg.2016.00527, PMID: 27148135 PMC4835762

[ref6] Babayiğit S. Stainthorp R. (2007). Preliterate phonological awareness and early literacy skills in Turkish. J. Res. Read. 30, 394–413. doi: 10.1111/j.1467-9817.2007.00350.x

[ref7] Babayiğit S. Stainthorp R. (2011). Modeling the relationships between cognitive–linguistic skills and literacy skills: new insights from a transparent orthography. J. Educ. Psychol. 103, 169–189. doi: 10.1037/a0021671

[ref8] Bird E. K. R. Cleave P. L. McConnell L. (2000). Reading and phonological awareness in children with down syndrome: a longitudinal study. Am. J. Speech Lang. Pathol. 9, 319–330. doi: 10.1044/1058-0360.0904.319

[ref9] Bird E. K. R. Cleave P. L. White D. Pike H. Helmkay A. (2008). Written and oral narratives of children and adolescents with down syndrome. J. Speech Lang. Hear. Res. 51, 436–450. doi: 10.1044/1092-4388(2008/032), PMID: 18367688

[ref10] Boudreau D. (2002). Literacy skills in children and adolescents with down syndrome. Read. Writ. 15, 497–525. doi: 10.1023/A:1016389317827

[ref11] Byrne A. MacDonald J. Buckley S. (2002). Reading, language and memory skills: a comparative longitudinal study of children with down syndrome and their mainstream peers. Brit. J. Educ. Psychol. 72, 513–529. doi: 10.1348/00070990260377497, PMID: 12495564

[ref12] Caravolas M. (2004). Spelling development in alphabetic writing systems: a cross-linguistic perspective. Eur. Psychol. 9, 3–14. doi: 10.1027/1016-9040.9.1.3

[ref13] Chilosi A. M. Brizzolara D. Lami L. Pizzoli C. Gasperini F. Pecini C. (2009). Reading and spelling disabilities in children with and without a history of early language delay: a neuropsychological and linguistic study. Child Neuropsychol. 15, 582–604. doi: 10.1080/09297040902927614, PMID: 19492202

[ref14] de Magalhães C. G. Cardoso-Martins C. Mervis C. B. (2022). Spelling abilities of school-aged children with Williams syndrome. Res. Dev. Disabil. 120:104129. doi: 10.1016/j.ridd.2021.104129, PMID: 34875548 PMC8724450

[ref15] Di Blasi F. D. Buono S. Città S. Costanzo A. A. Zoccolotti P. (2018). Reading deficits in intellectual disability are still an open question: a narrative review. Brain Sci. 8:146. doi: 10.3390/brainsci8080146, PMID: 30087288 PMC6119986

[ref16] Faust M. E. Balota D. A. Spieler D. H. Ferraro F. R. (1999). Individual differences in information-processing rate and amount: implications for group differences in response latency. Psychol. Bull. 125, 777–799. doi: 10.1037/0033-2909.125.6.777, PMID: 10589302

[ref17] Howlin P. Davies M. Udwin O. (1998). Cognitive functioning in adults with Williams syndrome. JCPP Adv. 39, 183–189. doi: 10.1111/1469-7610.00312 9669231

[ref18] Hulme C. Bowyer-Crane C. Carroll J. M. Duff F. J. Snowling M. J. (2012). The causal role of phoneme awareness and letter-sound knowledge in learning to read: combining intervention studies with mediation analyses. Psychol. Sci. 23, 572–577. doi: 10.1177/0956797611435921, PMID: 22539335

[ref19] Iaia M. Vizzi F. Rabuffetti M. Angelelli P. (2021). Lo studio dei processi lessicali e sublessicali di scrittura mediante l’esame dei parametri temporali in bambini di scuola primaria. G. Ital. Psicol. 48, 913–938. doi: 10.1421/104147

[ref20] Istituto Superiore di Sanità (2011). Consensus Conference, Disturbi Specifici dell’Apprendimento. Sistema nazionale per le linee guida Ministero della Salute. Roma, 6–7, Dicembre 2010.

[ref21] Johnson-Glenberg M. C. (2008). Fragile X syndrome: neural network models of sequencing and memory. Cogn. Syst. Res. 9, 274–292. doi: 10.1016/j.cogsys.2008.02.002, PMID: 19802322 PMC2577566

[ref22] Kent R. D. Vorperian H. K. (2013). Speech impairment in down syndrome: a review. J. Speech Lang. Hear. Res. 56, 178–210. doi: 10.1044/1092-4388(2012/12-0148), PMID: 23275397 PMC3584188

[ref23] Klusek J. Hunt A. W. Mirrett P. L. Hatton D. D. Hooper S. R. Roberts J. E. (2015). Reading and phonological skills in boys with fragile X syndrome. J. Autism Child. Schizophr. 45, 1699–1711. doi: 10.1007/s10803-014-2328-y, PMID: 25448919 PMC4442735

[ref24] Kortteinen H. Närhi V. Ahonen T. (2009). Does IQ matter in adolescents' reading disability? Learn. Individ. Differ. 19, 257–261. doi: 10.1016/j.lindif.2009.01.003

[ref25] Laing E. Hulme C. Grant J. Karmiloff-Smith A. (2001). Learning to read in Williams syndrome: looking beneath the surface of atypical reading development. JCPP Adv. 42, 729–739. doi: 10.1111/1469-7610.00769, PMID: 11583245

[ref26] Lavra-Pinto B. D. Lamprecht R. R. (2010). Phonological awareness and writing skills in children with down syndrome. Pró-Fono Revista de Atualização Científica 22, 287–292. doi: 10.1590/S0104-56872010000300022, PMID: 21103720

[ref28] Lewandowski K. E. Shashi V. Berry P. M. Kwapil T. R. (2007). Schizophrenic-like neurocognitive deficits in children and adolescents with 22q11 deletion syndrome. Am. J. Med. Genet. 144B, 27–36. doi: 10.1002/ajmg.b.30379, PMID: 17034021

[ref29] Lindström E. R. Lemons C. J. (2021). Teaching reading to students with intellectual and developmental disabilities: an observation study. Res. Dev. Disabil. 115:103990. doi: 10.1016/j.ridd.2021.103990, PMID: 34119889

[ref30] Loveall S. J. Conners F. A. (2016). Reading skills in down syndrome: an examination of orthographic knowledge. Am. J. Intellect. Dev. Disabil. 121, 95–110. doi: 10.1352/1944-7558-121.2.95, PMID: 26914465

[ref31] Luzzatti C. Colombo C. Frustaci M. Vitolo F. (2000). Rehabilitation of spelling along the sub-word level routine. Neuropsychol. Rehabil. 10, 249–278. doi: 10.1080/096020100389156

[ref32] Maehler C. Schuchardt K. (2016). The importance of working memory for school achievement in primary school children with intellectual or learning disabilities. Res. Dev. Disabil. 58, 1–8. doi: 10.1016/j.ridd.2016.08.007 27567244

[ref33] Marinelli C. V. Romani C. Burani C. Zoccolotti P. (2015). Spelling acquisition in English and Italian: a cross-linguistic study. Front. Psychol. 6:1843. doi: 10.3389/fpsyg.2015.01843, PMID: 26696918 PMC4672065

[ref34] McCarthy J. H. Hogan T. P. Catts H. W. (2012). Is weak oral language associated with poor spelling in school-age children with specific language impairment, dyslexia or both? J. Multiling. Commun. Disord. 26, 791–805. doi: 10.3109/02699206.2012.702185, PMID: 22876769 PMC3884899

[ref35] Menghini D. Verucci L. Vicari S. (2004). Reading and phonological awareness in Williams syndrome. Neuropsychology 18, 29–37. doi: 10.1037/0894-4105.18.1.29, PMID: 14744185

[ref36] Notarnicola A. Angelelli P. Judica A. Zoccolotti P. (2012). The development of spelling skills in a shallow orthography: the case of the Italian language. Read. Writ. 25, 1171–1194. doi: 10.1007/s11145-011-9312-0

[ref37] Orsini A. Pezzuti L. Picone L. (2012). WISC-IV: Contributo alla taratura Italiana [WISC-IV Italian Edition]. Florence, Italy: Giunti O. S.

[ref38] Pagon R. A. Bennett F. C. LaVeck B. Stewart K. B. Johnson J. (1987). Williams syndrome: features in late childhood and adolescence. Pediatrics 80, 85–91. doi: 10.1542/peds.80.1.85, PMID: 3601523

[ref39] Patterson K. (1986). Lexical but nonsemantic spelling? Cogn. Neuropsychol. 3, 341–367. doi: 10.1080/02643298608253363

[ref40] Pruneti C. Fenu A. Freschi G. Rota S. Cocci D. Marchionni M. (1996). Aggiornamento della standardizzazione italiana del test delle Matrici Progressive Colorate di Raven. Boll. Psicol. Appl. 217, 51–57.

[ref41] Ratz C. Lenhard W. (2013). Reading skills among students with intellectual disabilities. Res. Dev. Disabil. 34, 1740–1748. doi: 10.1016/j.ridd.2013.01.021 23500168

[ref42] Sprenger-Charolles L. Colé P. Serniclaes W. (2006). Reading Acquisition and Developmental Dyslexia, 1st Edn. London: Psychology Press.

[ref43] Swillen A. Vandeputte L. Cracco J. Maes B. Ghesquière P. Devriendt K. (1999). Neuropsychological, learning and psychosocial profile of primary school aged children with the velo-cardio-facial syndrome (22q11 deletion): evidence for a nonverbal learning disability? Child Neuropsychol. 5, 230–241. doi: 10.1076/0929-7049(199912)05:04;1-R;FT230, PMID: 10925707

[ref44] Tressoldi C. Cornoldi C. Re A. M., (2013). BVSCO-2. Batteria per la Valutazione della Scrittura e della Competenza Ortografica 2. Firenze: Giunti O.S.

[ref001] UNESCO Institute for Statistics (2012). International Standard Classification of Education ISCED 2011. UIS, Montreal, Quebec.

[ref45] Van den Broeck W. Geudens A. (2012). Old and new ways to study characteristics of reading disability: the case of the nonword-reading deficit. Cogn. Psychol. 65, 414–456. doi: 10.1016/j.cogpsych.2012.06.003, PMID: 22859020

[ref46] Varuzza C. De Rose P. Vicari S. Menghini D. (2015). Writing abilities in intellectual disabilities: a comparison between down and Williams syndrome. Appl. Res. Ment. Retard. 37, 135–142. doi: 10.1016/j.ridd.2014.11.011, PMID: 25463246

[ref47] Verucci L. Menghini D. Vicari S. (2006). Reading skills and phonological awareness acquisition in down syndrome. J. Ment. Defic. Res. 50, 477–491. doi: 10.1111/j.1365-2788.2006.00793.x, PMID: 16774633

[ref48] Vicari S. Bellucci S. Carlesimo G. A. (2001). Procedural learning deficit in children with Williams syndrome. Neuropsychologia 39, 665–677. doi: 10.1016/S0028-3932(01)00012-4, PMID: 11311297

[ref49] Vizzi F. Angelelli P. Iaia M. Risser A. H. Marinelli C. V. (2022). Writing composition ability and spelling competence in deaf subjects: a psycholinguistic analysis of source of difficulties. Read. Writ., 1–26. doi: 10.1007/s11145-022-10335-w

[ref50] Wechsler D. (2004). The Wechsler Intelligence Scale for Children-Fourth Edition. London, UK: Pearson Assessment.

[ref51] Wolff P. H. Melngailis I. Kotwica K. (1996). Family patterns of developmental dyslexia part III: spelling errors as behavioral phenotype. Am. J. Med. Genet. Suppl. 67, 378–386. doi: 10.1002/(SICI)1096-8628(19960726)67:4<378::AID-AJMG11>3.0.CO;2-G, PMID: 8837706

[ref52] Ziegler J. C. Pech-Georgel C. George F. Alario F. X. Lorenzi C. (2005). Deficits in speech perception predict language learning impairment. Proc. Natl. Acad. Sci. U. S. A. 102, 14110–14115. doi: 10.1073/pnas.0504446102, PMID: 16162673 PMC1236551

[ref53] Zoccolotti P. (2020). The reading level matched design: limitations and possible alternatives. Cogn. Neuropsychol. 37, 523–534. doi: 10.1080/02643294.2020.1809364, PMID: 32845816

[ref54] Zoccolotti P. De Luca M. Di Filippo G. Judica A. Martelli M. (2009). Reading development in an orthographically regular language: effects of length, frequency, lexicality and global processing ability. Read. Writ. 22, 1053–1079. doi: 10.1007/s11145-008-9144-8

